# Investigation of Carbon Nanotube Grafted Graphene Oxide Hybrid Aerogel for Polystyrene Composites with Reinforced Mechanical Performance

**DOI:** 10.3390/polym13050735

**Published:** 2021-02-27

**Authors:** Yanzeng Sun, Hui Xu, Zetian Zhao, Lina Zhang, Lichun Ma, Guozheng Zhao, Guojun Song, Xiaoru Li

**Affiliations:** Institute of Polymer Materials, School of Material Science and Engineering, Qingdao University, No. 308 Ningxia Road, Qingdao 266071, China; yz400765@163.com (Y.S.); xuhui15689987880@163.com (H.X.); tianjianliuxing@foxmail.com (Z.Z.); zln15689955265@163.com (L.Z.); mlc840311@163.com (L.M.); zhaoguozhengqdu@163.com (G.Z.)

**Keywords:** CNT-GO aerogel, in-situ polymerization, grafting reaction, mechanical properties, thermal conductivity

## Abstract

The rational design of carbon nanomaterials-reinforced polymer matrix composites based on the excellent properties of three-dimensional porous materials still remains a significant challenge. Herein, a novel approach is developed for preparing large-scale 3D carbon nanotubes (CNTs) and graphene oxide (GO) aerogel (GO-CNTA) by direct grafting of CNTs onto GO. Following this, styrene was backfilled into the prepared aerogel and polymerized in situ to form GO–CNTA/polystyrene (PS) nanocomposites. The results of X-ray photoelectron spectroscopy (XPS) and Raman spectroscopy indicate the successful establishment of CNTs and GO-CNT and the excellent mechanical properties of the 3D frameworks using GO-CNT aerogel. The nanocomposite fabricated with around 1.0 wt% GO-CNT aerogel displayed excellent thermal conductivity of 0.127 W/m∙K and its mechanical properties were significantly enhanced compared with pristine PS, with its tensile, flexural, and compressive strengths increased by 9.01%, 46.8%, and 59.8%, respectively. This facile preparation method provides a new route for facilitating their large-scale production.

## 1. Introduction

Due to their light weight and high performance, graphene oxide (GO)–carbon nanotube (CNT)-polymer nanocomposites have been widely studied and applied in various industries, including aerospace [1], military, and national defense, and some engineering materials [2,3,4]. Three-dimensional (3D) GO aerogels can act as carriers for CNTs by enabling them to disperse evenly in the wall of GO aerogel, followed by filling the matrix with a suitable polymer material, thereby overcoming poor dispersion of the nanomaterials within the polymer [5,6,7,8]. In previous studies, CNTs were dispersed in GO through physical mixing to form GO-CNT aerogels [9,10]. However, there have been a few systematic studies on the grafting of CNTs onto the surface of GO. Therefore, it is important to explore how the structure and state of the CNTs in the GO aerogel affect the properties of the nanocomposite [11].

In recent years, the use of 3D aerogel-reinforced filler has greatly improved the uneven dispersion of fillers and effectively enhanced the performance of the nanocomposites [12,13,14]. For instance, Fan et al. prepared graphene aerogels and filled them with poly(methyl methacrylate) (PMMA) to form nanocomposites; the mechanical properties of the nanocomposites were significantly enhanced compared to those prepared using the traditional blending and dispersion method. When the content of the graphene aerogels was increased from 0.67 to 2.50 vol.%, the microhardness of the nanocomposites increased from 303.6 to 462.5 MPa [15]. When the content of GO was 2 wt.%, the mechanical strength and elastic modulus of the GO/epoxy resin nanocomposites were increased by 50% and 19.6%, respectively, the glass transition temperature increased by 15.59 °C, and the thermal conductivity was 1.4 times higher than that of the neat epoxy resin [6]. In addition, the compressive strength and elastic modulus of 3D graphene aerogel-reinforced silicone rubber was higher than that of silicone rubber, with the hardness increasing from 9.2 HA for pure GO to 20.5 HA for the nanocomposite [7]. Li et al. grafted CNTs on GO to form a hybrid filler to be dispersed into an epoxy matrix. The results showed that the tensile modulus and the tensile strength were enhanced by ~36% and ~40%, respectively [16]. However, systematic studies on the influence of the GO and CNT morphologies and structures on the properties of nanocomposites produced by physical mixing and chemical grafting have not yet been conducted [17,18,19].

In this study, CNTs were grafted onto the surface of GO with a silane coupling agent KH550 (3-Aminopropyltriethoxysilane), and 3D large-scale aerogels were prepared by freeze-drying. A mixture of styrene and an initiator were backfilled into the prepared 3D aerogel and polymerized in situ to form GO-CNT aerogel polystyrene (GO-CNTA/PS) nanocomposites. Subsequently, the mechanical properties of GO-CNT aerogels and GO-CNTA/PS nanocomposites were investigated.

## 2. Experimental

### 2.1. Materials

Graphite powder (about 20 μm) was purchased from the Huayuan Chemical Co., Ltd. (Shanghai, China), plain CNTs (length: 5~15 μm, diameter: ~20 nm, purity: ~97%) were purchased from Shenzhen Nano Company (Shenzen, China), styrene was purchased from Chemical Reagent Co, Ltd. (Tianjin, China), and azobisisobutyronitrile (AIBN) was purchased from the Tianjin Damao Chemical Reagent Factory (Tianjin, China). γ-Aminopropyl triethoxysilane (KH-550), N-hydroxysuccinimide (NHS), and 1-ethyl-3-(3-dimethylaminopropyl) carbodiimide (EDC) were purchased from the Shanghai Machlin Biochemical Co, Ltd. (Shanghai, China). Carboxylated CNTs (oxygen content: ~6.4%) were fabricated by the Institute of Polymer Materials, Qingdao University, China.

### 2.2. Preparation of the GO-CNT Aerogel

GO was prepared via an improved Hummers’ method from graphite flakes. CNTs were oxidized with a mixture of sulfuric acid and nitric acid at 100 °C for 8 h, and then the oxidized CNTs were obtained by centrifuging, filtering, and freeze-drying [20]. Next, the obtained CNTs (0.059 g) were mixed with ethanol (2 ml) in deionized (DI) water (21 mL) by sonication for 1 h. The mixture was then transferred to the GO suspension liquid (8.7 mL) and dispersed by sonication for 3 h (the mass ratio of CNTs to GO was 3:7). Following this, the suspension was transferred into a mold and held at −25°C for 12 h and then freeze-dried for 48 h.

The preparation process of GO-CNT aerogel was divided into two steps. First, to prepare the modified CNT-KH550, a mixture of 90 mL ethanol, 10 mL DI water, and 2 mL KH550 in a three-necked flask was ultrasonically dispersed for 1 h. Following this, CNTs were added and the mixture was ultrasonically dispersed for 2 h, followed by stirring at 78 °C for 6 h [21,22]. Finally, the prepared mixture was washed with acetone and ethanol, and then freeze-dried.

**Figure 1:**
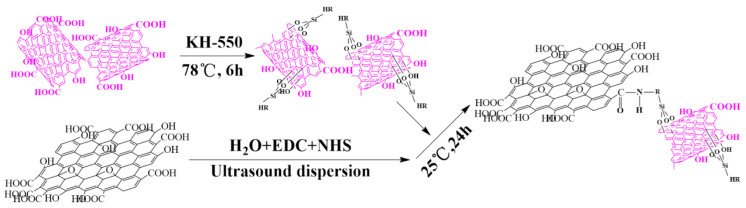
The reaction mechanism diagram.

Second, for the preparation of GO-CNT, GO, DI water, and ethanol were combined in a three-necked flask, after which ultrasonic dispersion was conducted for 3 h. Next, EDC/NHS (1:3) was added and the mixture stirred for 2 h at room temperature. Following this, the dispersed CNT-KH550 solution was added dropwise at 35 °C for 24 h. A schematic of the reaction mechanism is shown in Figure 1 [23,24,25].

### 2.3. Preparation of GO-CNTA/PS Nanocomposites

**Figure 2:**
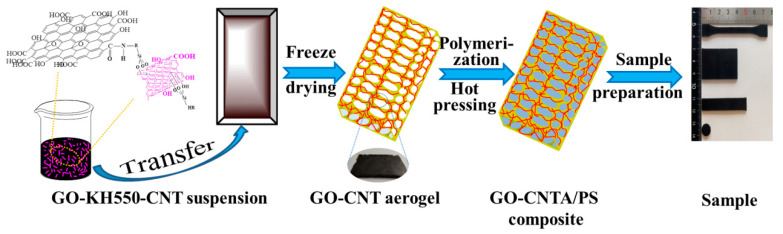
Preparation process of composite material.

The prepared GO-CNT aerogel was immersed in a mixture of styrene and AIBN via vacuum-assisted impregnation to ensure that the mixture replaced the air in the aerogel. The complexes were then maintained at 70 °C for 12 h, 80 °C for an additional 12 h, and 90 °C for another 12 h. The final GO-CNTA/PS nanocomposites were obtained after cooling and were molded by hot-pressing at 5 MPa and 170 °C for 5 min to discharge any tiny pores; the nanocomposites were only compressed by approximately 5% during the hot-pressing process. For comparison, a mixture of GO and CNTs (a total mass content of 1.0 wt.%, and a mass ratio of 7:3) was added to a pristine PS matrix to prepare blended nanocomposites, defined as GOC/PS. Abbreviation of the samples was shown in Table 1. The hot-pressing conditions shown in Figure 2 were the same for all samples.

### 2.4. Characterization

The synthesized GO-CNT morphology and chemical structure were analyzed via Fourier-transform infrared spectroscopy (FTIR; Nexus 360 ThermoNicolet, Singapore). Elemental and chemical compositions were evaluated by using X-ray photoelectron spectroscopy (XPS; Escalab 220i-XL, VG Systems, Ltd., East Grinstead, UK). GO and CNT structures were characterized by Raman spectroscopy (InVia 2000, Renishaw PLC, Wotton-Under-Edge, UK), and the structure and composition were analyzed via X-ray diffraction (XRD; D8 Advance, Bruker AXS GmBH, Karlsruhe, Germany) with Cu-Kα radiation and λ = 1.5418 Å. GO-CNT and nanocomposite morphologies were characterized by using field-emission scanning electron microscopy (FESEM; Hitachi S-5700, Hitachi High-Tech Analytical Science, Abingdon, UK) with gold for 30 s. Microhardness was measured with an HXD-1000TMC (Cany Precision Instruments Co., Ltd., Shanghai, China) digital microhardness tester using a 245 mN indentation force for 15 s.

Composite tensile, bending, impact, and compression tests were performed according to National Standards GB/T 1040.1-2006/ISO 527-1:1993, GB/T 9341-2008/ISO 178:2001, GB/T 1041-2008/ISO 604:2002, and GB/T 1043.1-2008/ISO 179-1:2000 using an a1-7000 tensile tester (GOTECH, Taiwan, China) over 0–30,000 N with 2 mm/min tensile test speed and a standard 25 mm sample-spacing. The test speed for the plastic bending test was 1 mm/min, with 32 mm sample-spacing. Compression tests were performed using the universal testing machine with a speed of 5 mm/min, and impact tests were performed using the universal testing machine with a simple-supported beam impact tester with 7.5 J impact energy and a 32 mm sample span.

## 3. Results and Discussion

### 3.1. Chemical Composition and Morphology of GO, CNT, CNT-KH550, and GO-CNT

**Figure 3:**
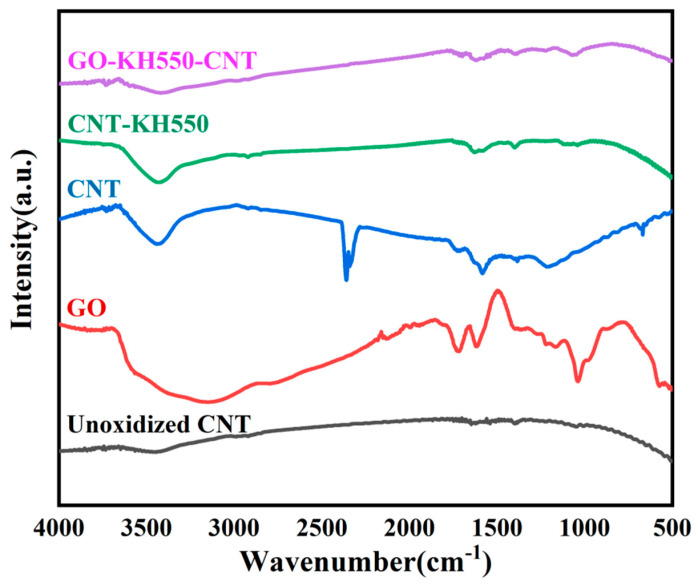
FTIR spectrum of GO, CNT, CNT-KH550 and GO-CNT. (color figure can be viewed online).

Figure 3 shows the structures and compositions of GO, CNTs, CNT-KH550, and GO-CNT determined via Fourier-transform infrared spectroscopy (FTIR). Absorption peaks for GO and CNTs were located at 3208 cm^−1^ (O-H tensile vibration), as well as 1721 and 1045 cm^−1^ (C=O and C-O tensile vibration, respectively), indicating that they contained a large number of hydroxyl and carboxyl groups. However, CNT-KH550 had an absorption peak at 1031 cm^−1^, which was attributed to Si-O-C tensile vibration, indicating that KH550 had been successfully grafted onto the surface of the CNTs. The peak intensity at 3208 cm^−1^ for GO-CNT decreased, indicating that the O-H groups had decreased significantly and CNT-KH550 had been successfully grafted onto the GO surface.

**Figure 4:**
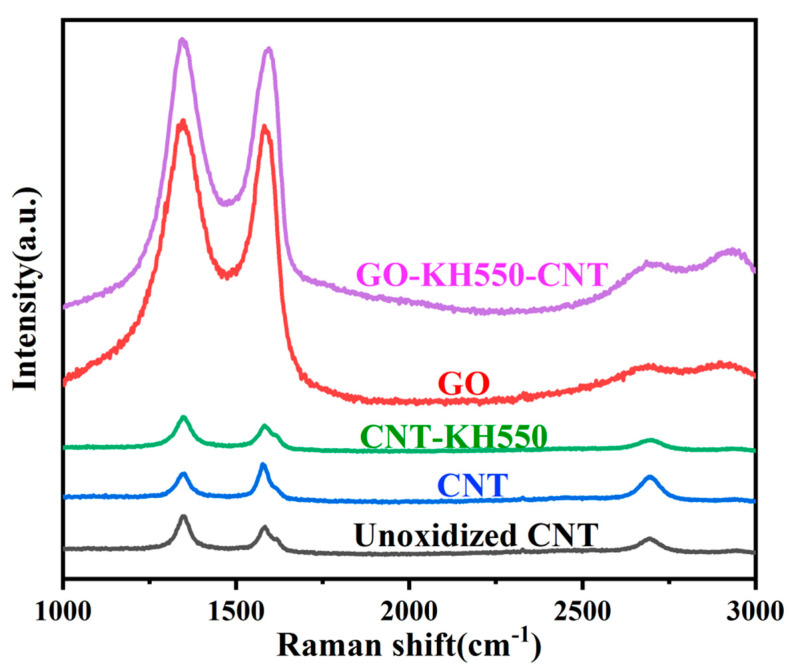
Raman spectroscopy (color figure can be viewed online).

Figure 4 shows Raman spectroscopy results for GO, CNTs, CNT-KH550, and GO-CNT. Two strong bands at 1351 (the D band) and 1594 cm^−1^ (the G band) correspond with the surface disorder and active groups of GO or CNT, respectively. It can be seen from the *I_D_*/*I_G_* ratio that the degree of disorder and reactivity of unoxidized CNTs (1.076), CNTs (1.270), and CNT-KH550 (1.466) gradually increased. Thus, the degree of disorder and surface activity for CNT-KH550 were high. Compared with GO (1.705), the degree of disorder and reactivity of GO-CNT (1.533) was reduced to a certain extent, which was due to the reaction of CNT-KH550 with the active groups on the GO surface, signifying that CNTs had been grafted onto the surface of the GO aerogel.

**Figure 5:**
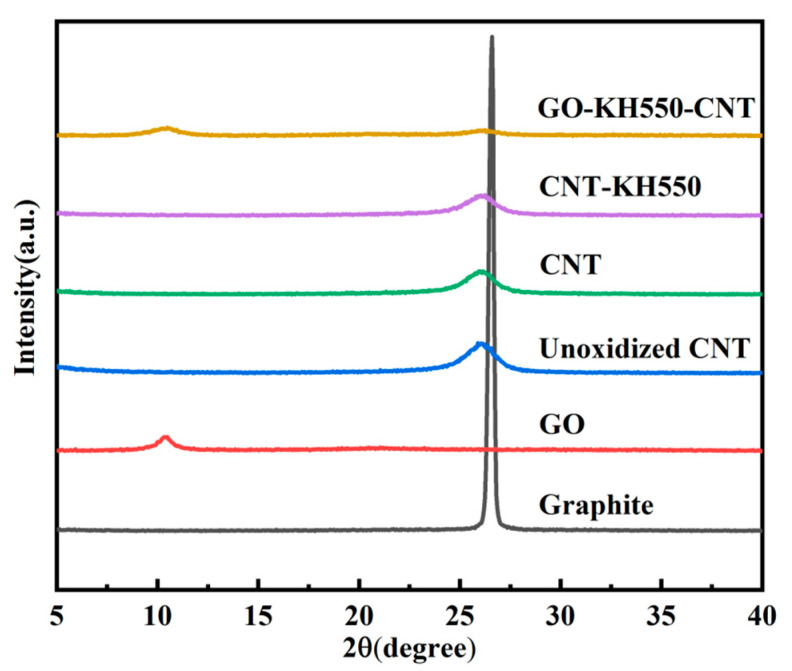
XRD patterns of Graphite, GO, unoxidized CNT, CNT, CNT-KH550 and GO-CNT.

Figure 5 shows XRD patterns of graphite, GO, unoxidized CNTs, CNTs, CNT-KH550, and GO-CNT. The diffraction peak at 26.51° corresponds to 0.34 nm interlayer spacing. GO had a large interlayer distance (~0.86 nm) due to hydroxyl and carboxyl group formation, indicating that graphite was successfully exfoliated during the chemical reaction. The diffraction peak at 25.95° for unoxidized CNTs, CNTs, and CNT-KH550 confirmed that the interlayer distances for these did not change because the hydroxyl and carboxyl groups do not affect the interlayer distance. However, the interlayer spacing of GO-CNT (~0.842 nm) was smaller than that of GO (~0.86 nm), suggesting that GO had reacted.

**Figure 6:**
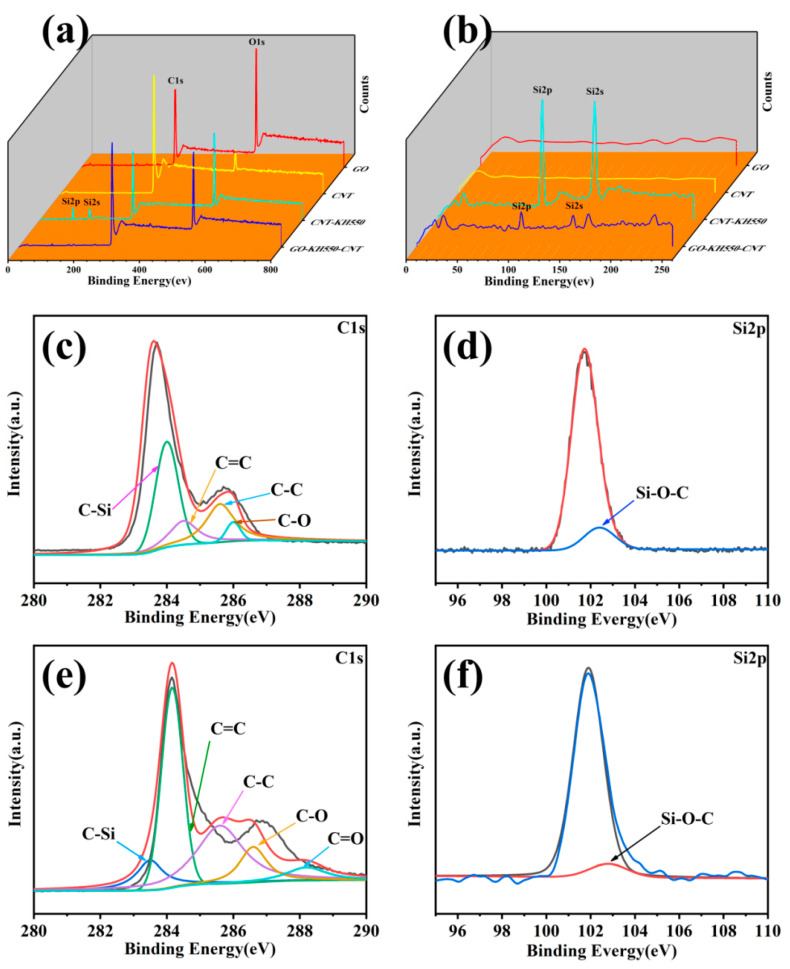
XPS spectra of GO, CNT, CNT-KH550 and CNT-GO, (**a**) and (**b**) XPS wide spectrum, (**c**) C1s spectrum of CNT-KH550, (**d**) Si2p spectrum of CNT-KH550, (**e**) C1s spectrum of GO-CNT, (**f**) Si2p spectrum of GO-CNT. (color figure can be viewed on line).

Figure 6 shows the surface chemical composition of GO, CNTs, CNT-KH550, and GO-CNT analyzed via XPS. In Figure 6a, the peaks at 285 and 530 EV correspond to C and O, respectively, in GO, CNTs, CNT-KH550, and GO-CNT. Figure 6b is an enlarged view of Figure 6a for the range of 0–250 eV. The peaks at 50 and 101 eV correspond to Si 2p and Si 2s, respectively, which helps to further indicate that KH550 and CNT-KH550 had been grafted onto the CNTs and GO, respectively. Figure 6c,d shows the C 1s regions of CNT-KH550 and GO-CNT, respectively, deconvoluted into six peaks, which were attributed to the binding energies of C-C (284.4 EV), C-O-C (286.5 eV), C=O (287.2 eV), C-OH (284.8 eV), and C (C=O)-OH (288.5eV), respectively. Figure 6d,f shows the Si 2p regions of CNT-KH550 and GO-CNT, respectively, especially the binding energy of Si-O-C at 1031 eV. Therefore, there were a lot of Si-O-C bonds present on the GO surface after the CNTs had been grafted.

### 3.2. Morphology and Dispersion of GO-CNT Aerogel and GO-CNTA/PS Nanocomposites

**Figure 7:**
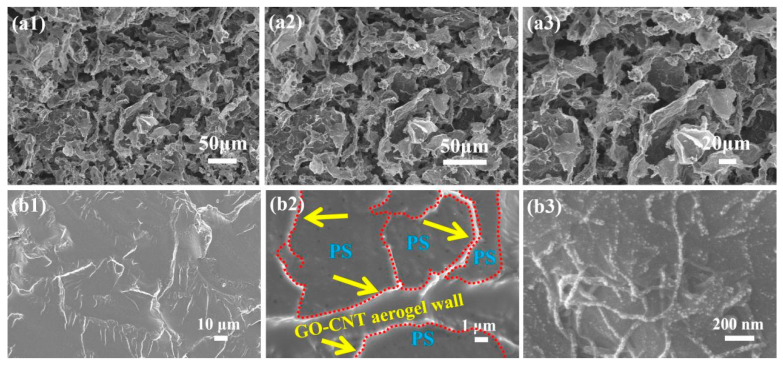
SEM images of (**a1**–**a3**) CNT-GO aerogels (**b1**–**b3**) the fractured surfaces of the CNT-GOA/PS.

The microstructure of the GO-CNT aerogel and GO-CNTA/PS nanocomposite were investigated via FE-SEM imaging, as shown in Figure 7. Figure 7a shows the GO-CNT aerogel images with different magnification, and Figure 7b exhibits nanocomposite fractured surface microstructures with different magnification. In Figure 7a, it can be seen that GO-CNT had a uniform 3D network, and the pores were interconnected with each other. However, the aerogel wall can be seen in the composite fractured surface as shown in Figure 7b1. The bright streaking is the wall of the aerogel, which was made of CNTs bonded to GO sheets. Seen from Figure 7b2, the red dotted lines show the walls of the aerogel, and the uniform parts between the walls are PS. Figure 7b3 shows the GO aerogel wall uniformly coated with CNTs; it can be clearly seen that the CNTs were only on the surface of GO and did not penetrate the GO sheet. A cross-section of GO-CNTA/PS (Figure 7b1) indicates that the matrix and reinforcement in the nanocomposites appear alternately, indicating that PS completely filled the pores in the aerogel.

**Figure 8:**
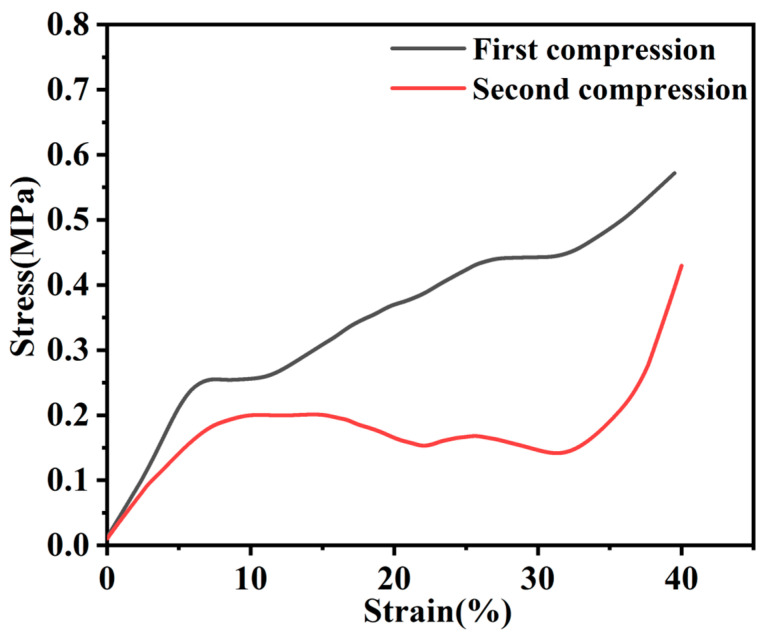
Stress-strain curves of compression for GO-CNT aerogel.

Figure 8 shows compression curves after two successive compressions of the columnar aerogel by 40% of its height. It can be seen that it bounced back naturally after the first compression with a compression strength of 0.26 MPa, and maintained a strength of 0.19 MPa after two successive compressions. This is due to the enhancement of the aerogel through the GO and CNTs being linked together.

### 3.3. Mechanical Properties of the Nanocomposites

**Figure 9:**
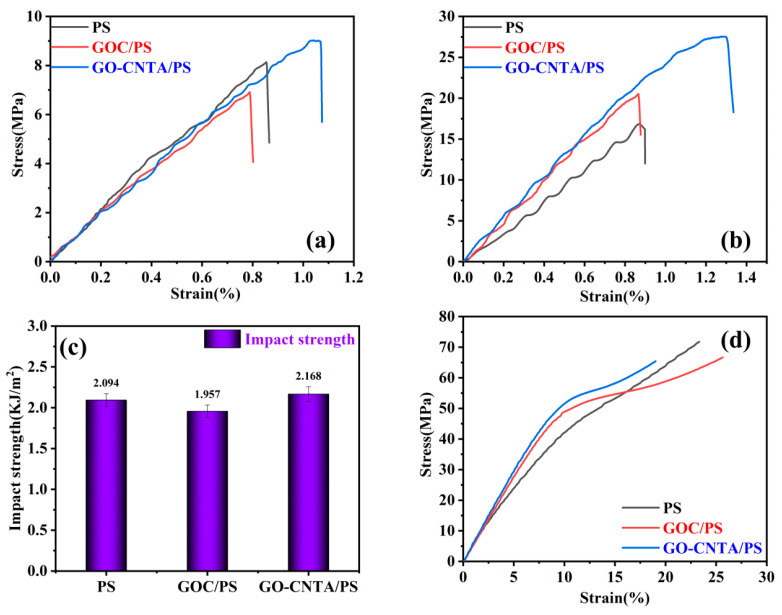
Mechanical properties of PS, GOC/PS, and GO-CNTA/PS: (**a**) typical stress-strain curves, (**b**) stress-strain curves of flexural, (**c**) impact strength, (**d**) stress-strain curves of compression. (color figure can be viewed online).

We explored the mechanical properties of pristine PS, GOC/PS, and GO-CNTA/PS using tensile, flexural, compressive, and impact measurements. Table 2 summarizes the various parameters for tensile strength, tensile strain, flexural strength, flexural strain, compressive strength, compressive strain, and impact strength. As shown in the stress–strain curves of the nanocomposites in Figure 9, we determined that the tensile, impact, and compressive strength values of the GO-CNTA/PS nanocomposites were improved by varying degrees compared with pristine PS: the tensile strength was improved by 9.01%, and that the bending strength was improved by 46.88%. Hence, the distribution of the CNTs in the GO aerogel and PS filling the aerogel pores had a direct effect on the mechanical properties of the nanocomposites.

**Figure 10:**
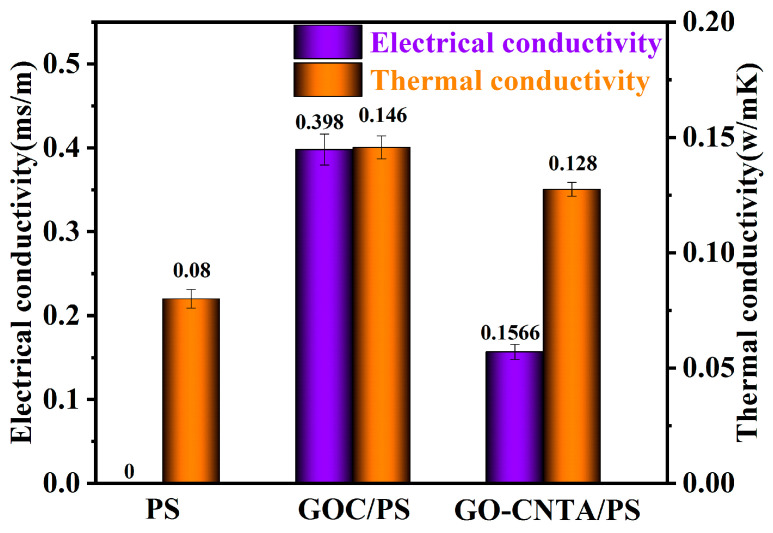
Electrical and thermal conductivity of neat PS, GOCA/PS and GO-CNTA/PS.

As shown in Figure 10, it can be seen that the electrical and thermal conductivity values of GO-CNTA/PS were 0.156 ms/m and 0.128 W/mK, respectively. Compared with pure PS with no electrical conductivity, the thermal conductivity increased by 60%. This is because the excellent electrical and thermal conductivity were enhanced by the CNTs being connected and evenly dispersed. However, the electrical and thermal conductivity values of GO-CNTA/PS were lower than those of GOC/PS. The possible reasons are as follows: (1) In the process of hot pressing, the structure of 3D aerogel may be damaged to a certain extent, resulting in lower electrical and thermal conductivity properties of the GO-CNTA/PS composite than that of the GOC/PS composite. (2) The grafting of carbon nanotubes onto the GO surface resulted in a decrease in the amount of CNTs between the GO sheets on the aerogel wall, and therefore the electrical and thermal conductivity of the GO-CNTA/PS composites was lower than that of the GOC/PS composites. The electrical and thermal conductivity of the composites is expected to be better if the CNT were doped in GO sheets. It also provides a reference and idea for the design of new composite materials.

**Figure 11:**
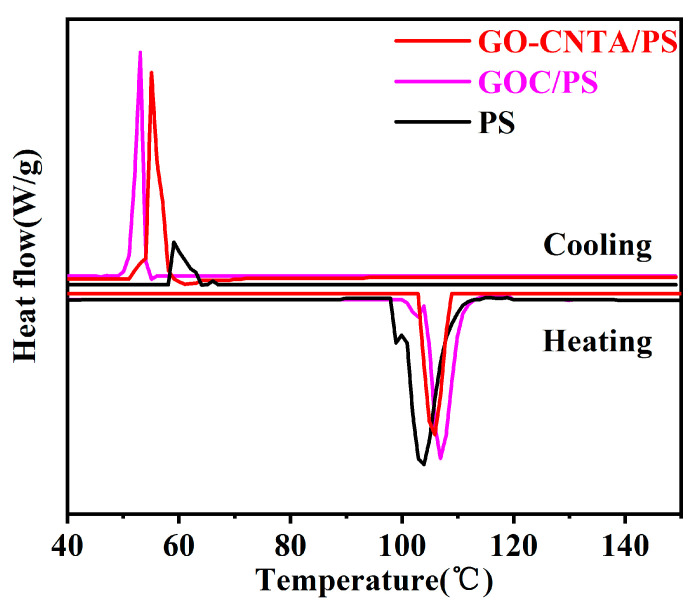
DSC curves of PS, GOC/PS and GO-CNTA/PS.

The thermal properties of the nanocomposites were also analyzed. Figure 11 shows the differential scanning calorimetry curves for the different samples. It can be seen that the glass transition temperature of GO-CNTA/PS was the highest, which was due to the physical crosslinking effect in the complete GO-CNT network in the nanocomposite. However, the 3D network structure for GO-CNT was incomplete and thus not able to improve the glass transition temperature of the nanocomposites. In addition, the crystallization tendency of polystyrene was enhanced by the induction of GO, and the crystallization temperature of GOCA/PS and GO-CNTA/PS composites was lower than that of pure polystyrene.

## 4. Conclusions

To the best of our knowledge, testing of standard splines has been rarely reported in the literature. In this work, we demonstrated a facile preparation method to fabricate a GO-CNT aerogel and GO-CNTA/PS nanocomposite on a large-scale and implemented standard spline cutting and testing. The surface energy of GO was reduced by surface modification, and the compatibility between inorganic materials and polymers was increased, thus improving the mechanical properties of the nanocomposites. The results show that the GO-CNT aerogel framework has high compression performance: the tensile, flexural, impact, and compressive strength of the obtained GO-CNTA/PS nanocomposites were significantly stronger than pristine PS, with increases of 9.01%, 46.8%, 3.73%, and 59.8%, respectively. Moreover, the thermal conductivity of the GO-CNTA/PS nanocomposites showed an increase of 60% to 0.128 W/m∙K compared to pristine PS.

To sum up, the innovative points of this work are as follows: (1) large-scale GO-CNT aerogel was prepared, (2) large-scale GO-CNTA/PS composite sheet was prepared via in situ polymerization, (3) realized the mechanical test of the standard sample.

## Figures and Tables

**Table 1:** Abbreviation of sample.

Full Name	Abbreviation
GO, CNT and polystyrene blending composite	GOC/PS
polystyren	PS
CNT covalently bonded to KH550	CNT-KH550
CNT grafted GO aerogel	GO-CNTA
GO-CNT aerogel/polystyrene composite	GO-CNTA/PS

**Table 2:** Mechanical properties of different composites.

Sample	Tensile Strength (MPa)	Tensile Strain (%)	Flexural Strength (MPa)	Flexural Strain (%)	Impact Strength (KJ/m^2^)	Compressive Strength (MPa)	Compressive Strain (%)
PS	8.29	0.858	18.74	0.95	2.09	33.45	7.56
GOC/PS	6.8	0.785	22.18	0.79	1.96	38.72	6.08
GO-CNTA/PS	9.037	1.074	27.525	1.405	2.168	53.441	10.853

## Data Availability

The data presented in this study are available on request from the corresponding author.
